# Proton Compared to X-Irradiation Induces Different Protein Profiles in Oral Cancer Cells and Their Derived Extracellular Vesicles

**DOI:** 10.3390/ijms242316983

**Published:** 2023-11-30

**Authors:** Inga Solgård Juvkam, Olga Zlygosteva, Mateusz Sitarz, Bernd Thiede, Brita Singers Sørensen, Eirik Malinen, Nina Jeppesen Edin, Tine Merete Søland, Hilde Kanli Galtung

**Affiliations:** 1 Institute of Oral Biology, Faculty of Dentistry, University of Oslo, 0372 Oslo, Norway; i.s.juvkam@odont.uio.no (I.S.J.); t.m.soland@odont.uio.no (T.M.S.); 2 Department of Radiation Biology, Institute for Cancer Research, Oslo University Hospital, 0379 Oslo, Norway; eirik.malinen@fys.uio.no; 3 Department of Physics, Faculty of Mathematics and Natural Sciences, University of Oslo, 0371 Oslo, Norway; olga.zlygosteva@fys.uio.no (O.Z.); n.f.j.edin@fys.uio.no (N.J.E.); 4 Danish Centre for Particle Therapy, Aarhus University Hospital, 8200 Aarhus, Denmark; mateusz.sitarz@fuwu.edu.pl (M.S.); bsin@oncology.au.dk (B.S.S.); 5 Department of Biosciences, Faculty of Mathematics and Natural Sciences, University of Oslo, 0371 Oslo, Norway; bernd.thiede@ibv.uio.no; 6 Department of Experimental Clinical Oncology, Aarhus University Hospital, 8200 Aarhus, Denmark; 7 Department of Pathology, Oslo University Hospital, 0372 Oslo, Norway

**Keywords:** protons, X-rays, oral squamous cell carcinoma, extracellular vesicles, non-targeted effects of radiation

## Abstract

Extracellular vesicles (EVs) are membrane-bound particles released from cells, and their cargo can alter the function of recipient cells. EVs from X-irradiated cells have been shown to play a likely role in non-targeted effects. However, EVs derived from proton irradiated cells have not yet been studied. We aimed to investigate the proteome of EVs and their cell of origin after proton or X-irradiation. The EVs were derived from a human oral squamous cell carcinoma (OSCC) cell line exposed to 0, 4, or 8 Gy from either protons or X-rays. The EVs and irradiated OSCC cells underwent liquid chromatography–mass spectrometry for protein identification. Interestingly, we found different protein profiles both in the EVs and in the OSCC cells after proton irradiation compared to X-irradiation. In the EVs, we found that protons cause a downregulation of proteins involved in cell growth and DNA damage response compared to X-rays. In the OSCC cells, proton and X-irradiation induced dissimilar cell death pathways and distinct DNA damage repair systems. These results are of potential importance for understanding how non-targeted effects in normal tissue can be limited and for future implementation of proton therapy in the clinic.

## 1. Introduction

Extracellular vesicles (EVs) are membrane-bound particles released from cells into the extracellular space. From their cell of origin, EVs carry nucleic acids, lipids and proteins that can alter the function of the recipient cells via uptake or membrane binding. Thus, EVs are important in inter-cellular communication in physiological as well as pathological processes [1,2,3].

Ionising radiation used in cancer therapy leads to DNA damage, which is the primary cause of subsequent cellular effects. Similar damage, termed non-targeted effects of radiation or radiation-induced bystander effects, can also be found in non-irradiated neighbouring or distant cells [4,5,6]. Recent research has shown that EVs from irradiated cells may be one of the delivery methods of non-targeted effects of radiation [7,8,9,10], which may have several implications for radiotherapy [11].

Studies on the role of EVs in non-targeted effects has, to our knowledge, only been performed using photon irradiation such as gamma and X-rays [12,13,14,15]. Protons deposit most of their energy in the so-called Bragg peak with no dose deposited deeper in the tissue [16,17]. This provides a clinical advantage over X-rays when reducing the dose received by normal tissue surrounding the tumour. Therefore, proton irradiation is expected to reduce the quantity of side effects in normal tissue compared to X-rays and is implemented in an increasing number of radiotherapy centres around the world. However, EVs derived from proton-irradiated cancer cells may still induce non-targeted effects in normal cells.

We hypothesise that protons may induce differential protein profiles in EVs compared to X-rays due to the difference in how the two types of radiation deposit energy in tissue. Protons have an elevated relative biological effectiveness (RBE) compared to X-rays [18,19,20]. RBE is defined as the ratio of a reference X-ray dose to the proton dose that induces the same biological effect. It depends on several variables, such as linear energy transfer (LET), which describes the amount of energy transferred from each ionising particle to the tissue per distance unit. LET and RBE are closely associated with each other, and high-LET radiation usually leads to stronger biological effects than low-LET radiation [21]. Accumulating research suggests that RBE increases at the distal end of the Bragg peak, where the LET is highest [22,23,24,25]. Therefore, it is of interest to investigate the differences between low- and high-LET protons as well as comparing protons to X-rays.

The protein content and possible effects of EVs on recipient cells derived from proton-irradiated cancer cells have, to our knowledge, not been investigated. The aim of this study was to investigate the proteome of EVs derived from a human oral squamous cell carcinoma (OSCC) cell line exposed to either protons or X-rays. Furthermore, the proteome of the EV-releasing OSCC cells after exposure to protons and X-rays was evaluated. Such knowledge may be important to our understanding of how non-targeted effects in normal tissue can be limited and for the future implementation of proton therapy in the clinic.

## 2. Results

### 2.1. EV Characterization and Overview of Protein Content

Nanoparticle tracking analysis (NTA), showed that the median EV size was approximately 200 µm, with sizes typically ranging from 60–250 nm, which was also confirmed with transmission electron microscopy (TEM) (Figure S1A,B). There were no significant differences in regard to the size of the EVs between the treatment modalities. X-rays gave a significantly higher EV concentration compared to the two proton treatment groups (high and low-LET) (Figure S1C). However, this variation in EV concentration between X-rays and protons was also seen in the corresponding non-irradiated controls and is therefore most likely due to variations in cell batch and ambient conditions, not because of the radiation treatment itself.

**Figure 1:**
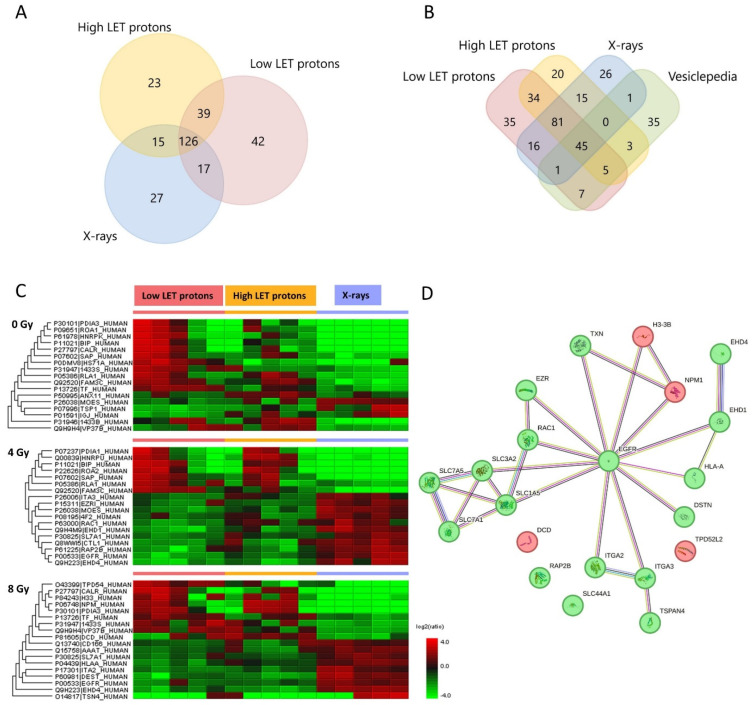
(**A**) Venn diagram of all proteins detected in EVs isolated from OSCC cells after exposure to low-LET protons, high-LET protons or X-rays, created in FunRich version 3.1.3. (**B**) Venn diagram of all detected EV proteins in our study compared to the 100 most common proteins detected in EVs according to the Vesiclepedia database, created in FunRich version 3.1.3. (**C**) Heat map of significantly (fold change ≥ 1, *p* < 0.05) up- (red) or downregulated (green) proteins in EVs after proton or X-irradiation (*n* = 5 in all treatment and dose groups). (**D**) STRING analysis of protein–protein network of all EV proteins found to be up- (red) or downregulated (green) after proton (high- and low LET with 4 and 8 Gy combined) compared to X-irradiation as seen from Table 1. Due to the small number of proteins significantly upregulated after proton vs. X-irradiation and the small difference seen between high- and low-LET protons, only one STRING analysis network is included for EV proteins.

Proteomic analysis of the EVs identified 224 proteins after low-LET proton irradiation, 203 proteins after high-LET proton irradiation, 185 proteins after X-irradiation and 186 proteins in non-irradiated controls (Figure 1A). Compared to the 100 most predominant proteins generally detected in EVs according to the Vesiclepedia database (microvesicles.org), the majority were also detected in the EVs in this study (Figure 1B and Table S1), including CD9 and CD81, which have been identified as typical EV markers [2]. This aids in confirming that the isolated particles were EVs. EV isolation and characterisation was performed while adhering to the MISEV guidelines [2,26,27].

### 2.2. Up- and Downregulated EV Proteins after Irradiation

Of the EV proteins identified in the EV samples, only some were significantly up- or downregulated compared to the other treatment groups (shown in the heat map in Figure 1C). However, of these significantly expressed proteins, some were also similarly expressed in the corresponding non-irradiated controls. Therefore, these proteins were suspected not to be upregulated by the radiation itself, and they were hence excluded from further analysis. After excluding proteins that were not different from the non-irradiated controls, the proteins shown in Table 1 were included as statistically significant EV proteins.

Proteomic analysis of the EVs isolated from proton-irradiated OSCC cells showed similar protein expression after high- and low-LET protons, except for three proteins; solute carrier family 7 member 5 (SLC7A5), thioredoxin (TXN) and Rac family small GTPase 1 (RAC1). Except for these three proteins, the protein profile in the EVs were the same after high- and low-LET protons. Therefore, in the further analysis of EV proteomics, the EV results from the high- and low-LET protons were combined. EV proteins downregulated after proton compared to X-irradiation were mostly related to cell adhesion, migration, amino acid transport, cell cycle and growth, programmed cell death and inflammatory response. EV proteins upregulated after proton compared to X-irradiation were related to cell proliferation, nucleosome assembly, cytoskeleton organization and immune system processes (Table 1).

In the protein–protein interaction network analysis, the epidermal growth factor receptor (EGFR), which was downregulated after exposure to protons compared to X-rays, functioned as a hub protein for all the other protein interaction networks (Figure 1D). Additionally, the transporter proteins solute carrier family 1 member 5 (SLC1A5), solute carrier family 3 member 2 (SLC3A2), solute carrier family 7 member 1 (SLC7A1), and solute carrier family 7 member 5 (SLC7A5) interacted with each other and formed a dense network. Moreover, EH domain-containing protein 1 (EHD1) and EH domain-containing protein 4 (EHD4) were also tightly connected with many interactions. EHD1 and EHD4 were downregulated after proton versus X-irradiation and EHD4 after both 4 and 8 Gy, while EHD1 only after 4 Gy.

### 2.3. Up- and Downregulated OSCC Cell Proteins after Irradiation

**Figure 2:**
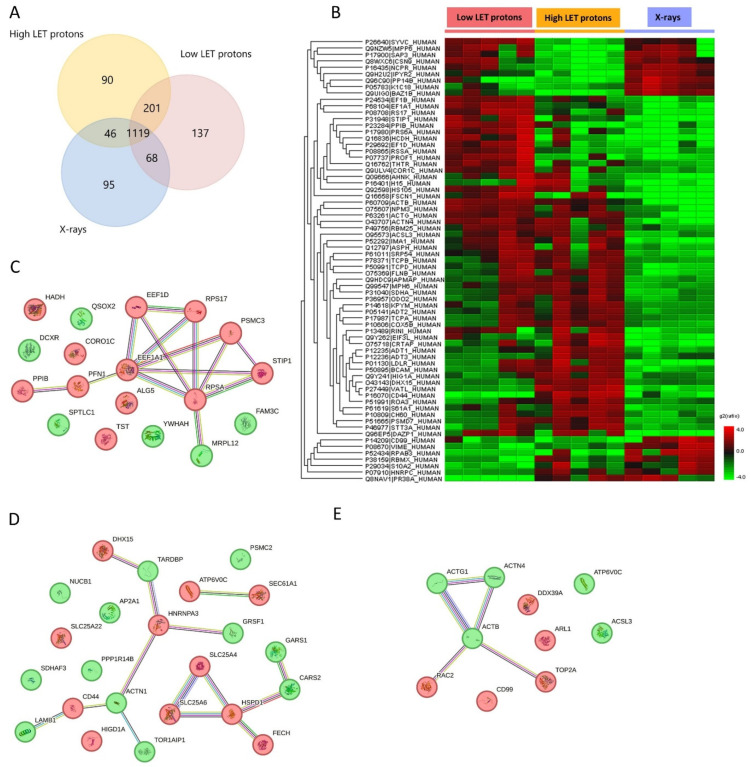
(**A**) Venn diagram of all proteins detected in OSCC cells after high- or low-LET proton or X-irradiation. Venn diagram created in FunRich version 3.1.3. (**B**) Heat map of significantly up- (red) and downregulated (green) proteins found in OSCC cells after 8 Gy irradiation with low- or high-LET protons or X-rays (fold change ≥ 2, *p* < 0.05). *n* = 5 in all treatment and dose groups. (**C**–**E**) STRING analysis of protein–protein network of all cell proteins found to be up- (red) or downregulated (green) after irradiation with (**C**) low-LET protons, (**D**) high-LET protons and (**E**) X-rays.

Proteomic analysis generally identified a larger number of proteins in OSCC cells than in their associated EVs after exposure to either proton or X-irradiation. After low-LET protons, 1525 proteins were detected in the OSCC cells, while 1456 proteins were identified after high-LET protons, 1328 proteins after X-rays and 1310 proteins in non-irradiated controls (Figure 2A). Differentially expressed proteins in the three treatment groups are presented in Figure 2B and Table 2 and Table 3.

Proteins that were upregulated in OSCC cells after irradiation with low-LET protons were related to cell migration or adhesion, DNA replication, transcription or translation and cell death, while downregulated proteins were related to epithelial–mesenchymal transition, regulation of ROS and programmed cell death (Table 2 and Table 3). Proteins upregulated in OSCC cells after irradiation with high-LET protons had functions associated with programmed cell death, regulation of DNA damage response and inflammatory response, while downregulated proteins were associated with cell migration or adhesion, RNA splicing, protein translation and programmed cell death (Table 2 and Table 3). Taken together, high-LET protons caused upregulation of proteins important in DNA damage response and programmed cell death, which is not seen after low-LET proton irradiation, indicating more complex cell damage after high-LET proton irradiation compared to low-LET proton irradiation. After irradiation with X-rays, OSCC cells showed upregulation of proteins related to cell adhesion, mRNA splicing and transport, DNA double-strand breaks, regulation of ROS and programmed cell death compared to high- and low-LET proton irradiation. On the other hand, proteins related to migration or angiogenesis, cell cycle and DNA repair, programmed cell death and lipid metabolism were downregulated after X-irradiation compared to high- and low-LET protons (Table 2 and Table 3).

Protein–protein networks using STRING analysis revealed several protein interactions (Figure 2C–E). Due to the larger number of proteins up- or downregulated in OSCC cells compared to their associated EVs, three separate STRING analysis networks are shown in Figure 2C–E, separated by treatment. After low-LET proton irradiation, most of the upregulated proteins were connected via protein–protein interactions. Here, elongation factor 1-alpha 1 (EEF1A1) functioned as a hub protein with many interactions with other upregulated proteins (Figure 2C). After high-LET proton irradiation, several separate protein interaction networks were identified (Figure 2D). The largest interaction network was one where several up- and downregulated proteins were connected to each other, many of which were involved in RNA splicing and apoptosis. A smaller network was identified, where the upregulated ADP/ATP translocases SLC25A4 and SLC25A6 connected to the upregulated 60 kDa heat shock protein HSPD1, all of which are involved in apoptosis. After X-irradiation, one protein interaction network was found, where downregulated actin beta (ACTB) functioned as a hub protein connected to 4 other proteins (Figure 2E). Here, ACTB involved in DNA double-strand break repair (homologous recombination (HR)) interacts with upregulated DNA topoisomerase 2 alpha (TOP2A) which makes DNA double-strand breaks. This increased double-strand breaks via TOP2A, but a decrease in DNA repair via HR. Taken together, this suggests that OSCC cells exposed to X-rays use an ACTB-independent pathway to HR, or non-homologous end joining (NHEJ), as the DNA repair pathway.

## 3. Discussion

EVs from irradiated cells have been proposed as one of the delivery vehicles of non-targeted effects of radiation, which may have several implications for radiotherapy [11]. Previous studies have shown that ionising radiation affects the cargo of EVs and, as a result, alter the function of the recipient cell by promoting migration and cell survival [12,28]. However, these studies have all been performed using gamma or X-rays. To our knowledge, the influence of proton irradiation on the cargo of EVs has not yet been studied. Therefore, the aim of our study was to investigate and compare the proteome of EVs derived from OSCC cells exposed to either protons or X-rays.

In the present study, proton irradiation resulted in downregulation of several solute carrier family proteins in the OSCC-derived EVs. Three of the downregulated solute carrier family proteins were SLC1A5, SLC7A5 and SLC3A2. The latter can form heterodimers with SLC7A11 and be involved in ferroptosis, which is a non-apoptotic form of cell death [29]. It can also produce heterodimers with SLC7A5 and be involved in the mTORC1 signaling pathway, important in the rapid growth of tumor cells [30]. Since a downregulation of both SLC3A2 and SLC7A5 were observed in EVs after proton irradiation, it is in the present study relevant to focus on the heterodimer between these two solute carrier proteins and its involvement in the mTORC1 pathway. It has previously been shown that upregulation of SLC3A2 in tumor biopsies was associated with poor survival of OSCC patients. Moreover, in vitro experiments with knockdown of SLC3A2 has been associated with reduced migration, invasion and proliferation and increased apoptosis of OSCC cells [31]. SLC3A2 has also been used as a cancer stem cell marker in head and neck squamous cell carcinoma [32]. Both the heterodimer SLC3A2/SLC7A5 and SLC1A5 play an important role in driving the uptake of glutamine and leucine, critical for metabolism and cellular function [30]. In the present study, proton irradiation compared to X-irradiation caused downregulation of all these three transporter proteins in EVs, potentially reducing the mTORC1 activation, which would negatively impact downstream effects of this pathway [33,34,35]. The main downstream pathway of mTORC1 is cell growth; however, recent research has also shown a link between mTORC1 and DNA damage response [36,37]. Here, mTOR-deficient cells showed a defect in recovering from the G2/M checkpoint after DNA damage. This may suggest that EVs derived from proton- rather than X-irradiated cells may reduce cell growth and DNA repair capability in recipient cells.

We observed an interesting downregulation of EGFR in EVs after proton irradiation, while it was upregulated after X-rays. EGFR is highly expressed in OSCC and has been documented to correlate with poor prognosis and resistance to radiation therapy [38,39,40]. Therefore, downregulation of EGFR in EVs derived from proton-irradiated OSCC cells could imply that protons are more efficient in non-targeted cell inactivation compared to X-rays. However, this needs to be further elucidated.

Our results show that several proteins preventing or negatively regulating apoptosis were upregulated in OSCC cells after irradiation with high-LET protons but were downregulated after irradiation with low-LET protons and X-rays. Only high-LET protons induced upregulation of the solute carrier family proteins SLC25A4 and SLC25A6 in OSCC cells, which are known to be involved in negative regulation of mitochondrial outer membrane permeabilisation leading to apoptosis [41,42]. In addition, HSPD1, both a positive and negative regulator of apoptosis [43,44], was upregulated after irradiation with high-LET protons. Of interest, a strong protein–protein interaction network with SLC25A4 and SLC25A6 was documented (Figure 2D), suggesting a negative regulation of apoptosis in this case. Moreover, HIGD1A has also been shown to prevent apoptosis via positive regulation of cytochrome c oxidase [45,46] and was upregulated in cells after exposure to high-LET protons. Lastly, SLC25A22 was also upregulated in cells after irradiation with high-LET protons and has been shown to promote proliferation and metastasis while inhibiting mitochondrial apoptosis via the MAPK/ERK pathway [47]. All these proteins preventing or negatively regulating apoptosis were upregulated after exposure to high-LET protons, but downregulated after exposure to low-LET protons and X-rays. Therefore, our results imply that high-LET protons could cause less apoptosis in OSCC cells compared to low-LET protons and X-rays. High-LET protons cause more clustered DNA damage because a larger amount of energy is transferred to the tissue, and therefore the chance of creating several double-strand breaks is increased [48,49,50]. Clustered DNA damage can be challenging for cells to repair, and more cell death could be expected. It has indeed been shown in lymphocytes that protons are more efficient in cell killing—not, however, primarily via apoptosis, but to a large degree via necrosis [51]. Thus, our results may suggest that even though high-LET protons seem to cause less apoptosis than X-rays, there could still be another type of cell death more prominent after proton irradiation. This is expected, as it has previously been shown in lung cancer cells that apoptosis only contributes to 5–10% of the total cell death after X-irradiation [52], while other types of cell death such as necroptosis and ferroptosis contribute to 8–10% and 14–18%, respectively, of the total cell death. Ferroptosis is a recently discovered type of cell death [53] which has gained interest in the last years. It is a non-apoptotic form of cell death depending on iron and the accumulation of lipid peroxides [54,55]. ACLS3 is known as an antiferroptotic protein [29], and ACSL3 was downregulated after X-irradiation, while it was upregulated after irradiation with both low- and high-LET protons. Therefore, it might seem that both high- and low-LET protons can cause less ferroptosis than X-rays in OSCC cells via upregulation of the antiferroptotic protein ACLS3. In summary, our results show that anti-apoptotic proteins were upregulated and an antiferroptotic protein was downregulated after proton irradiation compared to X-irradiation in OSCC cells. Taken together, this further supports the hypothesis that proton and X-irradiation induce different types of cell death.

ACTB is involved in positive regulation of double-strand break repair via HR [56,57]. Proton irradiation has previously been shown to increase the necessity for HR rather than NHEJ, potentially due to more clustered DNA damage compared to X-rays [48,58,59,60]. In the present study, ACTB was downregulated after irradiation with X-rays compared to protons. This might indicate that OSCC cells exposed to X-rays rather than protons use an ACTB-independent pathway for HR or use NHEJ as the DNA repair pathway. Taken together, this finding supports previous research which shows that proton and X-irradiation induce different types of DNA break repair systems.

## 4. Materials and Methods

### 4.1. Cell Irradiation

Human oral squamous cell carcinoma (PE/CA-PJ49/E10; ECACC; Salisbury, UK) cells were used in the present study. These cells have previously been specifically adapted to a low-serum vesicle-free medium for EV isolation to avoid contamination from fetal bovine serum [61] and been extensively studied in our lab [62,63,64]. Cells were seeded in T80 flasks at a concentration of 2.5 × 10^6^ cells in 15 mL of medium (Advanced DMEM + 1% exosome depleted FBS) in each flask. Cells were incubated for 48 h (until 70–80% confluent) at 37 °C with 5% CO_2_. Before irradiation, the cell flasks were completely filled with 200 mL of preheated and CO_2_-equilibrated medium. The flasks were irradiated in a vertical position as the fixed proton nozzle was horizontally aligned. Thus, a vertical position was used for both proton and X-irradiation. For each biological replicate, three T80 flasks were irradiated at the same time, and the culture medium pooled during the EV isolation process (described below) to ensure enough EVs per replicate.

Cells were irradiated with 6 MV X-rays using a linear accelerator at Oslo University Hospital (Varian Medical Systems, Palo Alto, CA, USA) or protons using a ProBeam system (Varian Medical Systems, Palo Alto, CA, USA) at the Danish Centre for Particle Therapy, Aarhus University Hospital, with doses of 0 Gy (sham irradiation), 4 Gy and 8 Gy (*n* = 5 for all treatment and dose groups). For proton irradiation, two different treatment plans were used: plan 1 (low-LET protons), where the cell flasks were placed at the entrance plateau of the pristine Bragg peak, where the LET is approximately 1 keV/µm; and plan 2 (high-LET protons) where the cell flasks were positioned at the distal end of the Bragg peak where the LET is approximately 12.4 keV/µm (Figure S2 and Table S2 for simulated LET values). Previously published values for clinical beams suggest an LET value of 0.2 keV/µm for 6 MV X-rays [65,66,67]. Immediately after irradiation, the medium was discarded, and 15 mL of fresh medium was added to ensure that the EVs to be isolated from the medium represented EVs derived from the irradiated cells and not EVs derived from the cells before and during irradiation and thereby becoming irradiated themselves. The conditioned medium was removed from the flasks 24 h after irradiation to perform EV isolation. After removal of the conditioned media, the irradiated cells were scraped off using a cell scraper and pooled into one 15 mL tube before being centrifuged at 1000× *g* for 5 min. Then the supernatant was removed and the cells were stored in PBS in −80 °C until proteomic analysis.

### 4.2. EV Isolation

**Figure 3:**
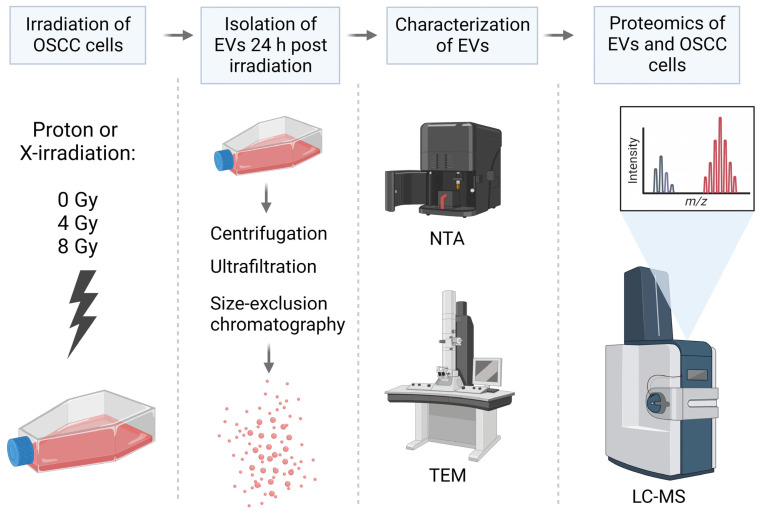
Graphical overview of the experimental design. Created with BioRender.com.

EVs were isolated from the media of irradiated E10 cells as previously described [61] (Figure 3). The media from each biological replicate were pooled into one tube, and the pooled samples were centrifuged at 4000× *g* for 5 min (Megafuge 1.0 R, Heraeus Instruments, Hanau, Germany) to remove cell debris. Then the supernatant was transferred to an empty tube and centrifuged at 15,000× *g* for 45 min (Centrifuge 5810 R, Eppendorf, Hamburg, Germany). Furthermore, the supernatant was concentrated via ultrafiltration using Amicon-Ultra columns (Merck Millipore, Tullagreen, Cork, Ireland) with a molecular cutoff of 100 kDa. Filter units were centrifuged at 4000× *g* for 5 min for each 15 mL of sample until the entire sample volume was reduced to only 50–200 µL. Thereafter, each concentrated sample was loaded onto a size-exclusion chromatography (SEC) column (IZON qEVoriginal, 70 nm, IZON Science, Christchurch, New Zealand) to separate the particles based on size. The eluate was collected into 16 sequential fractions of 0.5 mL. The protein concentration of each fraction was measured by spectrophotometry (Absorbance 280 nm, NanoDropOne, Thermo Fisher Scientific, Waltham, MA, USA), and the first 4 protein-rich fractions known from experience to contain EVs were pooled (fractions 7–10). The pooled EV samples were stored at −80 °C until proteomic analysis.

### 4.3. EV Characterization

Particle concentration and size distribution was analysed via nanoparticle tracking assay (NTA) on a NanoSight NS500 using NTA 3.4. EV morphology and size was further assessed using transmission electron microscopy (Philips CM120 BioTwin, Philips, Eindhoven, The Netherlands). The presence of characteristic EV markers was confirmed by comparing the 100 most common EV proteins according to the Vesiclepedia database (microvesicles.org) with the EV proteomic analysis (described below). We have submitted all relevant data of our experiment to the EV-TRACK knowledgebase (EV-TRACK ID: EV230980) [68].

### 4.4. Proteomic Analysis

#### 4.4.1. In-Solution Digestion

Pooled EV samples and cell pellets were thawed on ice, and 200 µL SILAC Phosphoprotein lysis buffer A and B (Invitrogen, Oslo, Norway) was added. The cell pellet was homogenized with a pestle (20×) for mechanical breakage of the cells and incubated for 10 min on ice. After this, the pooled EV samples and cell samples followed the same protocol. The samples were centrifuged at 2400× *g* for 10 min at 4 °C (Centrifuge 5415R, Eppendorf, Hamburg, Germany), and the supernatant was transferred to a new tube. Next, the proteins were precipitated by adding four volumes of ice-cold acetone, vortexed and incubated at −20 °C overnight. Subsequently, the samples were centrifuged at 16,000× *g* for 20 min at 4 °C (Centrifuge 5415R, Eppendorf, Hamburg, Germany), and the supernatant was discarded. The pellets containing the precipitated proteins were dissolved in 50 µL 6 M urea and 100 mM ammonium bicarbonate, pH 7.8. For reduction and alkylation of cysteines, 2.5 µL of 200 mM DTT in 100 mM Tris-HCl, pH 8 was added, and the samples were incubated at 37 °C for 1 h, followed by addition of 7.5 µL 200 mM iodoacetamide for 1 h at room temperature in the dark. The alkylation reaction was quenched by adding 10 µL 200 mM DTT at 37 °C for 1 h. Subsequently, the proteins were digested with 10 µg trypsin GOLD (Promega, Madison, WI, USA) for 16 h at 37 °C. The digestion was stopped by adding 5 µL 50% formic acid, and the generated peptides were purified using a 10 µL OMIX C18 micro-SPE pipette tip (Agilent, Santa Clara, CA, USA) and dried using a Speed Vac concentrator (Concentrator Plus, Eppendorf, Hamburg, Germany).

#### 4.4.2. LC-MS Analysis

The samples were analysed by LC-MS using a timsTOF Pro (Bruker Daltonics, Bremen, Germany) which was coupled online to a nanoElute nanoflow liquid chromatography system (Bruker Daltonics, Bremen, Germany) via a CaptiveSpray nanoelectrospray ion source. The dried peptides were dissolved in 4 µL 0.1% formic acid and 2 µL of sample was injected. The peptides were separated on a reversed phase C18 column (25 cm × 75 µm, 1.5 µm, PepSep (Bruker Daltonics, Bremen, Germany). Mobile phase A contained water with 0.1% formic acid, and acetonitrile with 0.1% formic acid was used as mobile phase B. The peptides were separated by a gradient from 0–35% mobile phase B over 60 min at a flow rate of 300 nL/min at a column temperature of 50 °C. MS acquisition was performed in DDA-PASEF mode. The capillary voltage was set to 1.5 kV with a mass range of 100 to 1700 m/z. The number of PASEF ranges was set to 20 with a total cycle time of 1.16 s, charge up to 5, target intensity of 20,000, intensity threshold of 1750 and active exclusion with release after 0.4 min. An inversed reduced TIMS mobility (1/*k*_0_) of 0.85–1.40 Vs/cm^2^ was used, with a range time of 100 ms, an accumulation time of 100 ms, a duty cycle of 100% and a ramp rate of 9.51 Hz. Precursors for data-dependent acquisition were fragmented with an ion mobility-dependent collision energy, which was linearly increased from 20 to 59 eV.

#### 4.4.3. Database Search

The LC/MS data were searched against the human Uniprot database (20,431 entries), with PEAKS X+ software version 10.5 (Bioinformatics Solutions, Waterloo, ON, Canada). The following parameters were used: digestion enzyme, trypsin; maximum missed cleavage, 2; fragment ion mass error tolerance, 0.03 Da; parent ion error tolerance, 15 ppm. Oxidation of methionine, and acetylation of the N-terminus were specified as variable modifications and carbamidomethylation of cysteines as a fixed modification. The maximum number of PTMs was set to 2. A false-discovery rate of 1% was applied to the datasets.

#### 4.4.4. Label-Free Quantitation

For label-free quantification (LFQ) using PEAKS, ID directed LFQ with outlier removal was applied. The following parameters were used on peptide features: quality ≥ 5, peptide ID count per group ≥1, detected in at least one sample per group. The following parameters were applied on protein: significance ≥20, fold change ≥1 (≥2 for the cell samples), significance method ANOVA with at least 2 peptides, and TIC was used for normalization of the data.

### 4.5. Protein Analysis

Venn diagrams were created in FunRich version 3.1.3 using all detected proteins in the treatment groups in EVs or OSCC cells. Proteins found to be significantly up- or downregulated in the corresponding non-irradiated controls were excluded in further analysis. Also, proteins that were up- or downregulated in fewer than 4 replicates were excluded from further analysis. All included statistically significant EV proteins are shown in Table 1 and all included cell proteins in Table 2 and Table 3. Protein–protein interaction networks were created in STRING version 12.0 using all significantly up- or downregulated proteins in the treatment groups in EVs or OSCC cells.

## 5. Conclusions

In the present study, we investigated the proteome of EVs derived from OSCC cells exposed to either protons or X-rays. Interestingly, we found differential protein profiles both in the EVs and in the OSCC cells after proton irradiation compared to X-irradiation. We observed a downregulation of EV proteins affecting cell growth and DNA damage repair after proton but not X-irradiation. In the OSCC cells, our results demonstrate that proton and X-irradiation induce distinct types of cell death as well as dissimilar DNA break repair systems. These results are of potential importance for understanding how non-targeted effects in normal tissue can be limited and for future implementation of proton therapy in the clinic.

## Figures and Tables

**Table 1:** EV proteins significantly up- or downregulated after proton irradiation (high- and low-LET) compared to X-irradiation (fold change > 1, *p* < 0.05). No EV proteins were significantly upregulated after 4 Gy of protons compared to X-rays.

Downregulated after 4 Gy		Downregulated after 8 Gy		Upregulated after 8 Gy	
Gene Name	Function	Gene Name	Function	Gene name	Function
ITGA3	Cell adhesion and migration, regulator of TGF- and Wnt signalling	ITGA2	Cell adhesion and migration, inflammatory response	TPD52L2	Carbohydrate metabolic processes, cell proliferation
SLC7A1	Amino acid transport, T-cell proliferation	SLC7A1	Amino acid transport, T-cell proliferation	H3-3B	Nucleosome assembly, cell growth regulation
SLC7A5 *	Immune system processes, programmed cell death, mTOR pathway	SLC7A5 *	Immune system processes, programmed cell death, mTOR pathway	NPM1	Programmed cell death, cytoskeleton organization
SLC3A2	RNA and protein binding, ferroptosis regulation, mTOR pathway	SLC1A5	Amino acid transport, ferroptosis regulation, mTOR pathway	DCD	Immune system processes, found in sweat
SLC44A1	Transmembrane transport, choline transport	HLA-A	Adaptive immune response, T-cell mediated cytotoxicity		
TXN *	Response to radiation, negative regulation of cell death,	TXN *	Response to radiation, negative regulation of cell death		
EGFR	Mitotic cell cycle, DNA repair, programmed cell death	EGFR	Mitotic cell cycle, DNA repair, programmed cell death		
EHD4	Endocytosis, endosomal transport, growth factor response	EHD4	Endocytosis, endosomal transport, growth factor response		
EHD1	Endocytosis, intracellular protein transport	DSTN	Cell motility, actin binding		
EZR	Immune system process, cytoskeleton organization	TSPAN4	Integral component of plasma membrane, focal adhesion		
RAC1 **	Inflammatory response, MAPK pathway, migration and proliferation				
RAP2B	Negative regulation of cell migration				

* Downregulated after irradiation with low-LET protons but not after high-LET protons. ** Downregulated after irradiation with high-LET protons, but upregulated after low-LET protons. These were the only EV proteins that were significantly different after low and high-LET protons. Proteins: Integrin subunit alpha 3 (ITGA3), solute carrier family 7 member 1 (SLC7A1), solute carrier family 7 member 5 (SLC7A5), solute carrier family 3 member 2 (SLC3A2), solute carrier family 44 member 1 (SLC44A1), thioredoxin (TXN), epidermal growth factor receptor (EGFR), EH domain-containing protein 4 (EHD4), EH domain-containing protein 1 (EHD1), ezrin (EZR), Rac family small GTPase 1 (RAC1), Ras-related protein Rab-2b (RAP2B), integrin subunit alpha 2 (ITGA2), solute carrier family 1 member 5 (SLC1A5), major histocompatibility complex, class 1, A (HLA-A), destrin (DEST), tetraspanin 4 (TSPAN4), TPD52 like 2 (TPD52L2), histone H3.3 (H3-3B), nucleophosmin 1 (NPM1), dermcidin (DCD).

**Table 2:** Upregulated OSCC cell proteins after proton compared to X-irradiation. Significantly different expressed proteins are presented (fold change > 2, *p* < 0.05). Dose groups 4 and 8 Gy were combined in this table as they showed the same tendency, only stronger in 8 Gy.

Low LET Protons		High LET Protons		X-rays	
Gene Name	Function	Gene Name	Function	Gene Name	Function
ALG5	Protein glycosylation	ATP6V0C	Autophagy, Wnt pathway	ARL1	Vesicle-mediated transport
CORO1C	Cell migration, endosomal transport	CD44	Inflammatory response, regulation of DNA damage response and apoptosis (p53)	CD99	Cell–cell adhesion
EEF1A1	Translation, EGF response	DHX15	RNA splicing, regulation of Ikb/NF-κB signalling	DDX39A	mRNA splicing and transport
EEF1D	Translation, cell death, cellular response to radiation	HIGD1A	Regulation of apoptotic process (hypoxia-induced protein), stress response	TOP2A	Makes ds DNA breaks, essential during mitosis and meiosis
HADH	Lipid metabolism	SEC61A1	Integral component of ER-membrane	RAC2	Regulation of apoptosis, augments the production of ROS
PPIB	RNA binding, positive regulation of organism growth	FECH	Detection of and response to UV light, heme biosynthesis, ferrous iron binding		
PFN1	Cell migration	HSPD1	Immune response, apoptosis		
PSMC3	DNA replication, transcription	HNRNPA3	mRNA splicing and transport		
RPS17	Translation	SLC25A22	Mitochondrial glutamate/H^+^ transporter		
RPSA	Translation, cell adhesion	SLC25A4	Regulation of mitochondrial membrane permeability (apoptosis)		
STIP1	Response to IL-7, HSP90 protein binding	SLC25A6	Regulation of mitochondrial membrane permeability (apoptosis)		
TST	Epithelial cell differentiation				

Abbreviations: EGF = epidermal growth factor; IL-7 = interleukin 7; ds = double-stranded; ROS = reactive oxygen species; HSP90 = heat shock protein 90. Proteins: dolichyl-phosphate beta-glucosyltransferase (ALG5), coronin-1C (CORO1C), elongation factor 1-alpha 1 (EEF1A1), elongation factor 1-delta (EEF1D), hydroxyacyl-coenzyme A dehydrogenase mitochondrial (HADH), peptidyl-prolyl cis-trans isomerase B (PPIB), profilin-1 (PFN1), 26S proteasome regulatory subunit 6A (PSMC3), small ribosomal subunit protein eS17 (RPS17), small ribosomal subunit protein uS2 (RPSA), stress-induced phosphoprotein 1 (STIP1), thiosulfate sulfurtransferase (TST), V-type proton ATPase 16 kDa proteolipid subunit c (ATP6V0C), CD44 antigen (CD44), ATP-dependent RNA helicase DHX15 (DHX15), HIG1 domain family member 1A, mitochondrial (HIGD1A), protein transport protein Sec61 subunit alpha isoform 1 (SEC61A1), ferrochelatase, mitochondrial (FECH), 60 kDa heat shock protein, mitochondrial (HSPD1), heterogenous nuclear ribonucleoprotein A3 (HNRNPA3), solute carrier family 25 member 22 (SLC25A22), solute carrier family 25 member 4 (SLC25A4), solute carrier family 25 member 6 (SLC25A6), ADP-ribosylation factor-like GTPase 1 (ARL1), DExD-box helicase 39A (DDX39A), DNA topoisomerase 2-alpha (TOP2A), Rac family small GTPase 2 (RAC2).

**Table 3:** Downregulated OSCC cell proteins after proton compared to X-irradiation. Significantly different expressed proteins are presented (fold change > 2, *p* < 0.05). Dose groups 4 and 8 Gy in each treatment group were combined in this table as they showed the same tendency, only stronger in 8 Gy.

Low LET Protons		High LET Protons		X-rays	
Gene Name	Function	Gene Name	Function	Gene Name	Function
FAM3C	Promotes epithelial to mesenchymal transition	GRSF1	RNA splicing and processing	ATP6V0C	Autophagy, Wnt pathway
DCXR	Regulation of ROS metabolic process	TARDBP	RNA splicing, apoptosis, cell cycle	ACTB	Cell cycle, DNA repair (HR), apoptosis
MRPL12	Mitochondrial translation, regulation of transcription	ACTN1	Apoptosis, transcription, cytoskeletal organization	ACTG1	Angiogenesis, gene expression, migration, response to INF-y
QSOX2	Protein folding, regulates sensitization of cells for INF-γ induced apoptosis	AP2A1	Endocytosis, intracellular protein transport	ACTN4	Migration, apoptosis, response to hypoxia
SPTLC1	Lipid metabolism, programmed cell death, inflammatory response	CARS2	Protein translation	ACSL3	Antiferroptotic, lipid metabolism
YWHAH	Regulation of apoptosis, transcription	GARS1	Protein translation		
		LAMB1	Cell adhesion, migration and proliferation		
		NUCB1	Small GTPase-mediated signal transduction		
		PSMC2	Cell differentiation, protein degradation		
		PPP1R14B	Innate immune response		
		SDHAF3	Mitochondrion organization		
		TOR1AIP1	Membrane organization		

Abbreviations: ROS = reactive oxygen species; INF-γ = Interferon gamma; HR = homologous recombination. Proteins: FAM3 metabolism regulating signalling molecule C (FAM3C), dicarbonyl and L-xylulose reductase (DCXR), mitochondrial ribosomal protein L12 (MRPL12), sulfhydryl oxidase 2 (QSOX2), serine palmitoyltransferase 1 (SPTLC1), tyrosin 3-monooxygenase protein eta (YWHAH), G-rich sequence factor 1 (GRSF1), TAR DNA-binding protein (TARDBP), alpha actinin 1 (ACTN1), adaptor related protein complex 2 subunit alpha 1 (AP2A1), cysteinyl-tRNA synthetase 2, mitochondrial (CARS2), glycyl-tRNA synthetase 1 (GARS1), Laminin subunit beta-1 (LAMB1), Nucleobindin-1 (NUCB1), 26S proteasome regulatory subunit 7 (PSMC2), protein phosphatase 1 regulatory inhibitor subunit 14B (PPP1R14B), succinate dehydrogenase complex assembly factor 3 (SDHAF3), torsin 1A interactin protein 1 (TOR1AIP1), V-type proton ATPase 16 kDa proteolipid subunit c (ATP6V0C), actin beta (ACTB), actin gamma 1 (ACTG1), actinin alpha 4 (ACTN4), acyl-CoA synthetase long chain family member 3 (ACSL3).

## Data Availability

All relevant data of our experiment has been submitted to the EV-TRACK knowledgebase (EV-TRACK ID: EV230980) [68].

## Supplementary Materials

The supporting information can be downloaded at: https://www.mdpi.com/article/10.3390/ijms242316983/s1.
