# Supplementary material for: Proton Compared to X-Irradiation Induces Different Protein Profiles in Oral Cancer Cells and Their Derived Extracellular Vesicles
Source: Int J Mol Sci. 2023 Nov 30;24(23):16983. doi: 10.3390/ijms242316983 (PMC10707519; doi:10.3390/ijms242316983)
Supplement: Supplementary file 1 [file ijms-24-16983-s001.zip › ijms-2697175-supplementary.pdf]

Supplementary materials

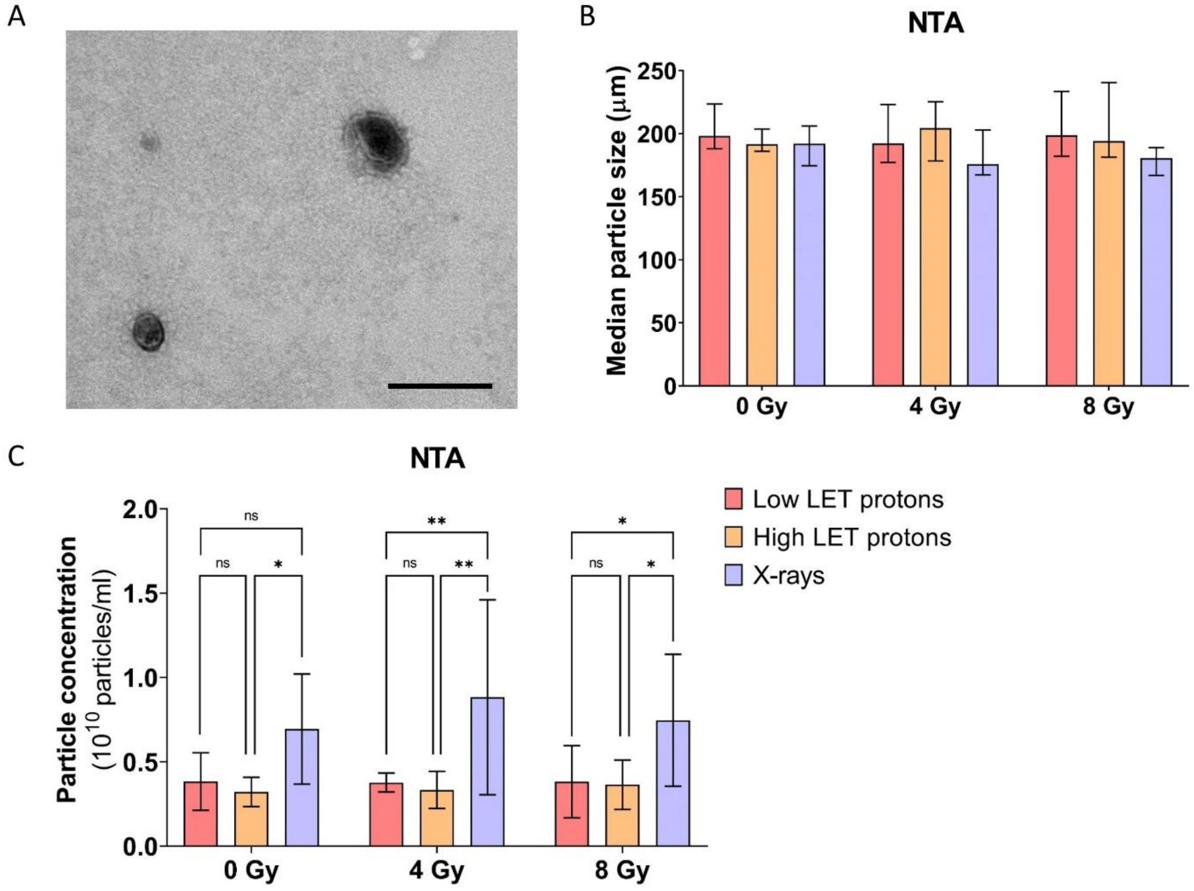

Figure S1. EV characterisation by particle concentration, size, and morphology using (A) negative staining in transmission electron microscopy, nanoparticle tracking analysis characterising the particles based on (B) median size and (C) particle concentration. Data are presented as mean ± 95 % CI. Asterisks are shown between groups that demonstrate statistically significant differences through two-way ANOVA. * < 0.05, ** ≤ 0.01, ns = not significant. Scale bar is 200 nm.

Table S1. Common EV proteins detected in the EV samples of each treatment group and the measured significance (log2(ratio)). Based on the 100 most common EV proteins from the Vesiclepedia database (microvesicles.org). Both dose groups (4 and 8 Gy) from the same treatment group were combined in this table. nd = not detected.

Vesiclepedia (Uniprot protein name)	Non-irradiated	Low LET protons	High LET protons	X-rays
1433B	20.88	0.35	13.3	11.3
1433E	4.50	0.40	2.09	2.91
1433T	11.60	10.9	10.48	2.29
1433Z	1.57	6.64	1.13	2.97
4F2	0.68	4.42	2.87	10.52
A2MG	5.70	0.81	0.91	nd
ACTB	6.70	2.55	0.09	0.10

ACTN4	11.22	1.86	nd	nd
ALBU	nd	2.14	4.67	3.70
ALDOA	4.79	0.28	0.49	1.36
ANXA1	0.19	1.13	3.52	0.29
ANXA11	20.49	0.09	4.25	1.53
ANXA2	2.15	0.74	0.92	1.14
ANXA5	2.11	0.10	1.44	0.39
ANXA7	0.88	nd	4.67	nd
AT1A1	3.68	3.98	1.92	7.42
BASI	2.51	1.42	6.11	4.01
BIP	35.61	0.38	1.09	nd
CD81	3.48	0.07	1.69	0.16
CD9	4.68	10.42	1.66	1.05
CDC42	14.68	0.91	nd	1.60
CLH1	3.02	1.54	0.89	5.95
CLIC1	2.93	1.23	0.75	0.34
COF1	6.89	7.14	4.1	6.26
EF1A1	6.39	1.38	0.84	1.70
EF2	9.05	1.14	3.22	0.01
ENOA	2.03	2.49	3.16	0.31
EZRI	3.91	2.76	4.54	3.54
G3P	16.77	2.99	11.42	2.14
GBB1	1.94	1.52	1.72	3.00
GBB2	6.87	nd	nd	3.82
GNAI2	5.34	5.95	4.7	2.37
H4	2.61	0.71	1.29	0.45
HS90A	2.45	1.56	1.99	nd
HS90B	nd	0.42	1.90	nd
HSP7C	17.30	2.22	3.53	8.25
IF4A1	7.05	3.36	2.35	nd
IMB1	nd	2.2	nd	nd
IQGA1	nd	0.52	nd	nd
ITB1	6.72	4.5	4.91	0.64
K1C10	16.04	2.71	2.03	0.26
K2C1	1.78	6.66	2.89	1.77
KPYM	17.99	1.3	0.64	4.90
LDHA	15.54	2.7	6.31	4.05
LDHB	nd	4.35	nd	nd
LG3BP	6.34	0.59	15.25	0.14
MOES	27.35	5.78	1.14	2.30
PDC6I	10.02	1.17	4.46	0.31
PPIA	3.57	1.90	1.99	1.03
PRDX1	nd	nd	0.29	nd
PROF1	7.83	1.62	7.76	0.19
RAB10	9.72	1.74	4.28	1.21
RAB5C	3.24	0.82	0.66	2.40
RAB7A	5.86	1.14	4.92	9.38
RAC1	7.80	3.49	4.45	3.50
RALA	2.81	0.16	nd	nd
RHOA	1.49	0.24	1.35	7.12
SDCB1	19.73	0.59	8.5	2.34
TCPB	nd	1.38	nd	nd

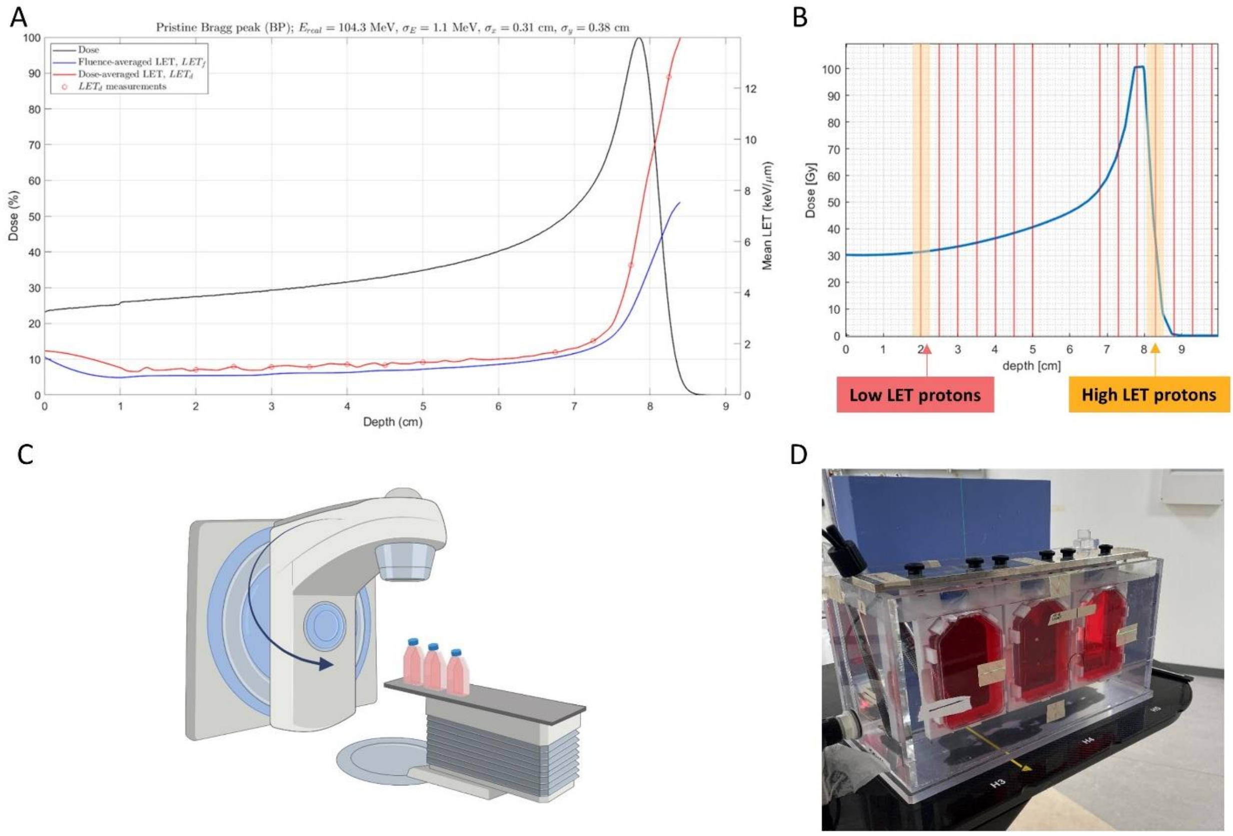

Figure S2. (A) Bragg peak in our experimental setup with dose (black line) and linear energy transfer (LET) (red line) where the LET increases at the distal end of the Bragg peak. (B) The two different chosen positions for proton irradiations shown in the Bragg peak from A. Position 1 is in the entrance plateau of the pristine Bragg peak where the LET is approximately 1 keV/µm, while position 2 is at the distal end of the Bragg peak where the LET is approximately 12.4 keV/µm. See Table S1 for simulated LET-values. (C) X-irradiation set-up. (D) Proton irradiation set-up.

Table S2. Simulated LET-values. Chosen positions for irradiation was at depth 2 cm and 8.25 cm (highlighted in bold).

Depth (cm)	*LETd (keV/µm)*
**2**	**0.9878**
2.5	1.1082
3	1.1007
3.5	1.1011
4	1.1977
4.5	1.1675
5	1.2812
6.75	1.6725
7.25	2.1148
7.75	5.0812
**8.25**	**12.4439**
